# Supplementary figures and images for: A crowdsourced set of curated structural variants for the human genome
Source: PLoS Comput Biol. 2020 Jun 19;16(6):e1007933. doi: 10.1371/journal.pcbi.1007933 (PMC7329145; doi:10.1371/journal.pcbi.1007933)

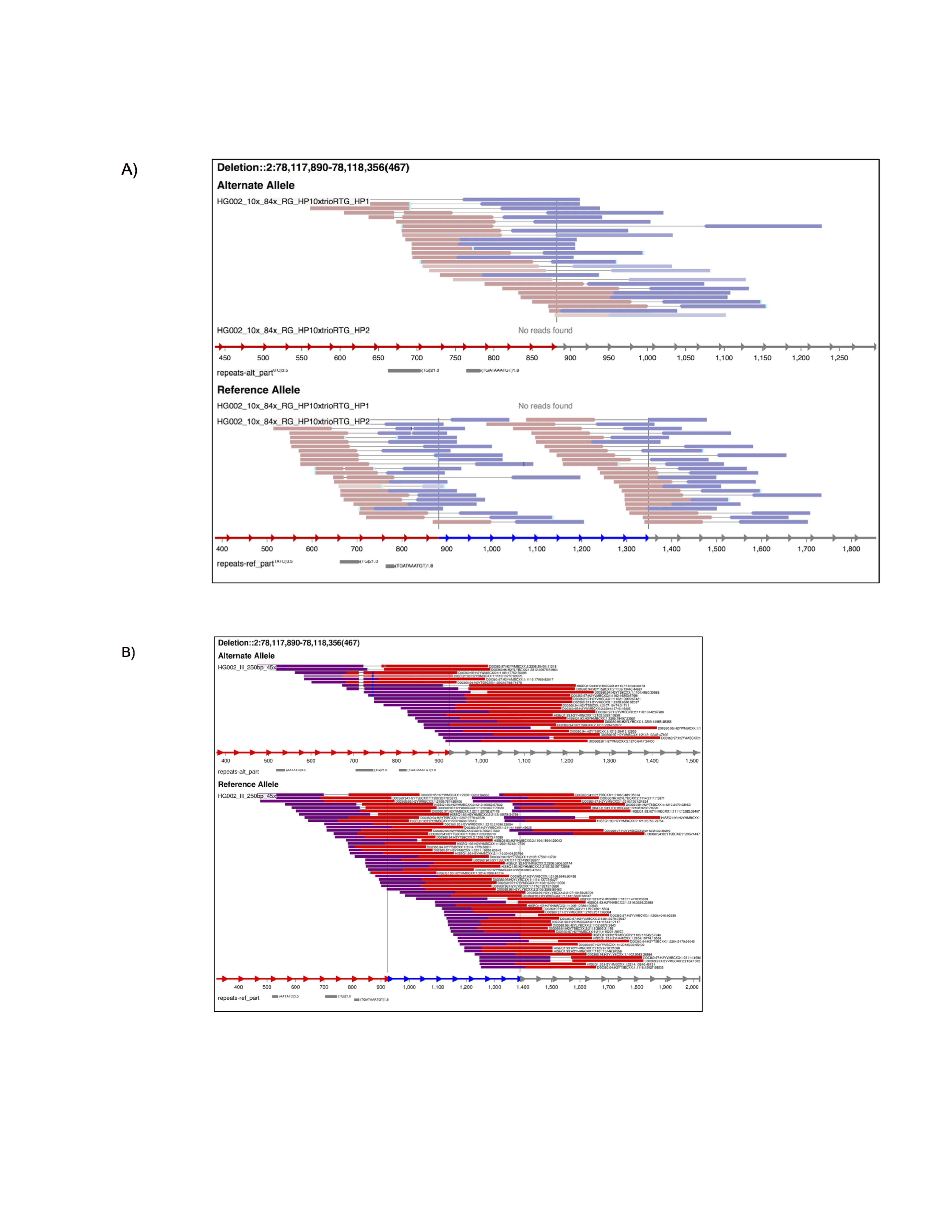

Supplement: S1 Fig — A putative 467bp deletion is shown. Svviz2 generates read aligned images for each short read and long read sequencing technology. A) svviz2 read aligned image - 10x Genomics (read length = 98bp; read depth = 50x). Reads were aligned to reference and alternate allele by svviz2. B) svviz2 read aligned image—Illumina HiSeq (read length = 250 bps; read depth = 40-50x). C) svviz2 read aligned image—Illumina HiSeq (read length = 148 bps; read depth = 296.83x). D) svviz2 read aligned image—Illumina Mate Pair (read length = 100 bps; insert size = 6000bp; read depth = 13-14x). E) svviz2 read aligned image—Haplotype separated PacBio (read length = 10-11kb; read depth = 69x). Reads were haplotype separated using WhatsHap and aligned to reference and alternate allele by svviz2. F) Svviz2 dotplot displaying reference versus alternate allele. G) Svviz2 dotplot PacBio read with the highest mapping quality score versus the alternate allele. H) IGV image showing reads aligned to a putative variant. IGV tracks include: Haplotype separated PacBio reads, Haplotype Separated 10x Genomics reads, Illumina 250x250bp paired end sequencing, and tracks to describe repeat regions (low complexity repeats and segmental duplications). All reads were aligned to GRCh37 human reference genome. I) Svviz2 dotplot displaying a putative deletion in a tandem repeat. (TIF) [file pcbi.1007933.s001.tif]

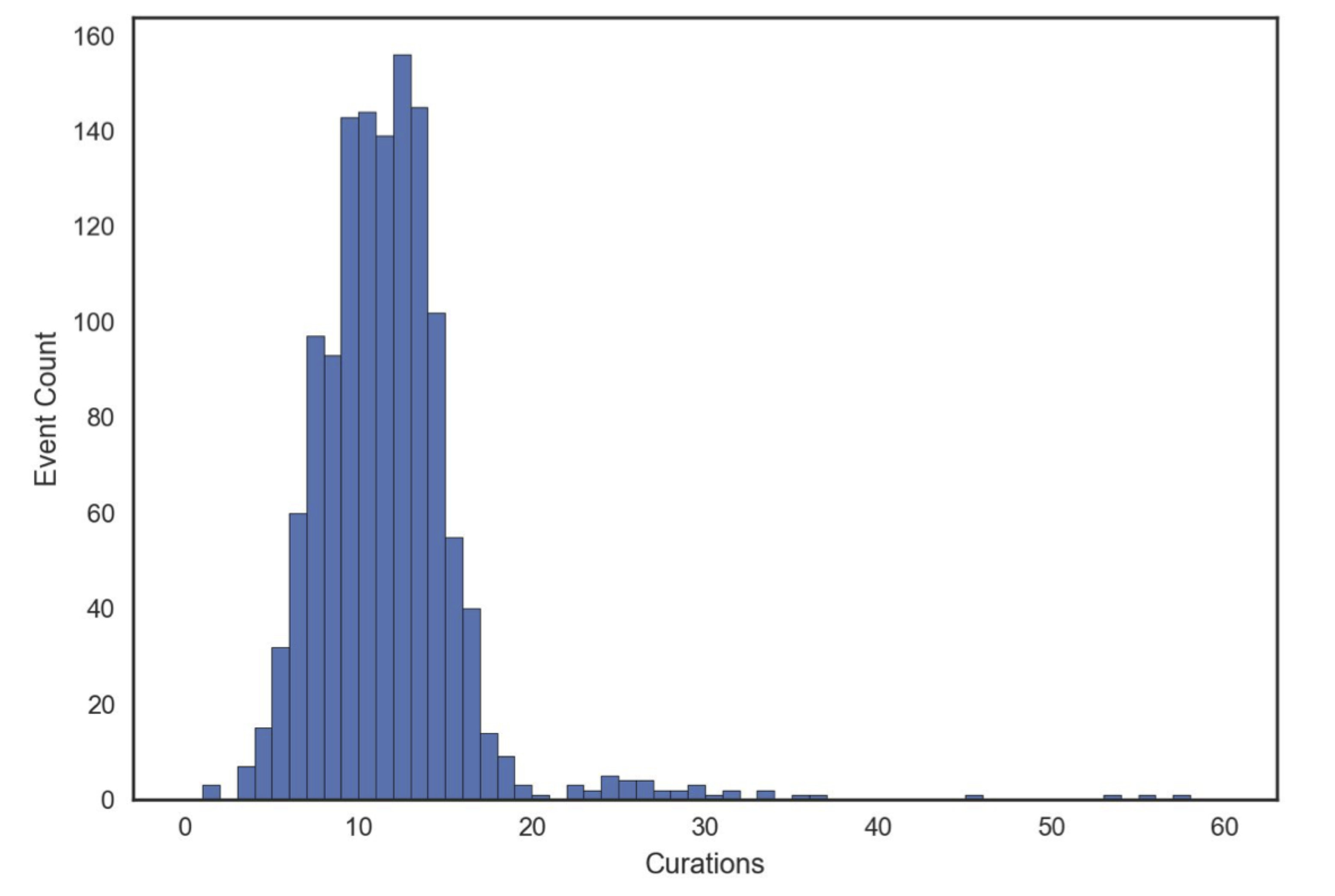

Supplement: S2 Fig — 61 curators evaluated SVCurator events. Each of the 1295 sites were curated on average 11 times with 1290 events curated at least 3 times. (TIF) [file pcbi.1007933.s002.tif]

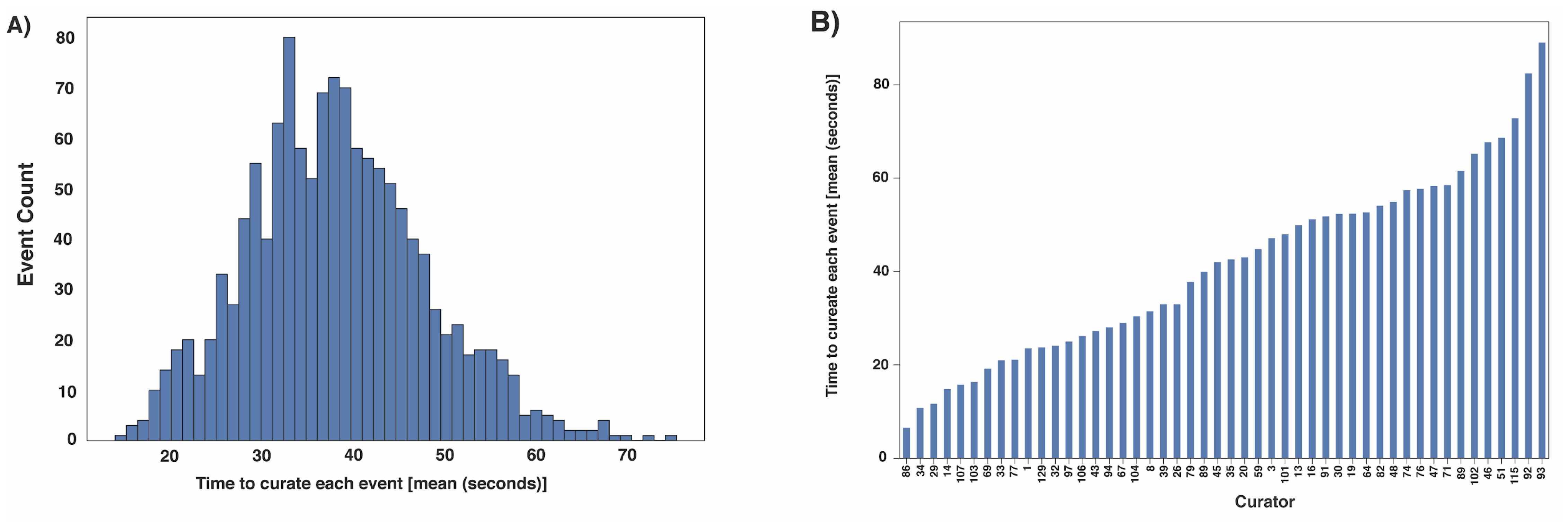

Supplement: S3 Fig — A) Overall distribution of the average time to curate events. B) Distribution of the average time to curate each event for each curator. (TIF) [file pcbi.1007933.s003.tif]

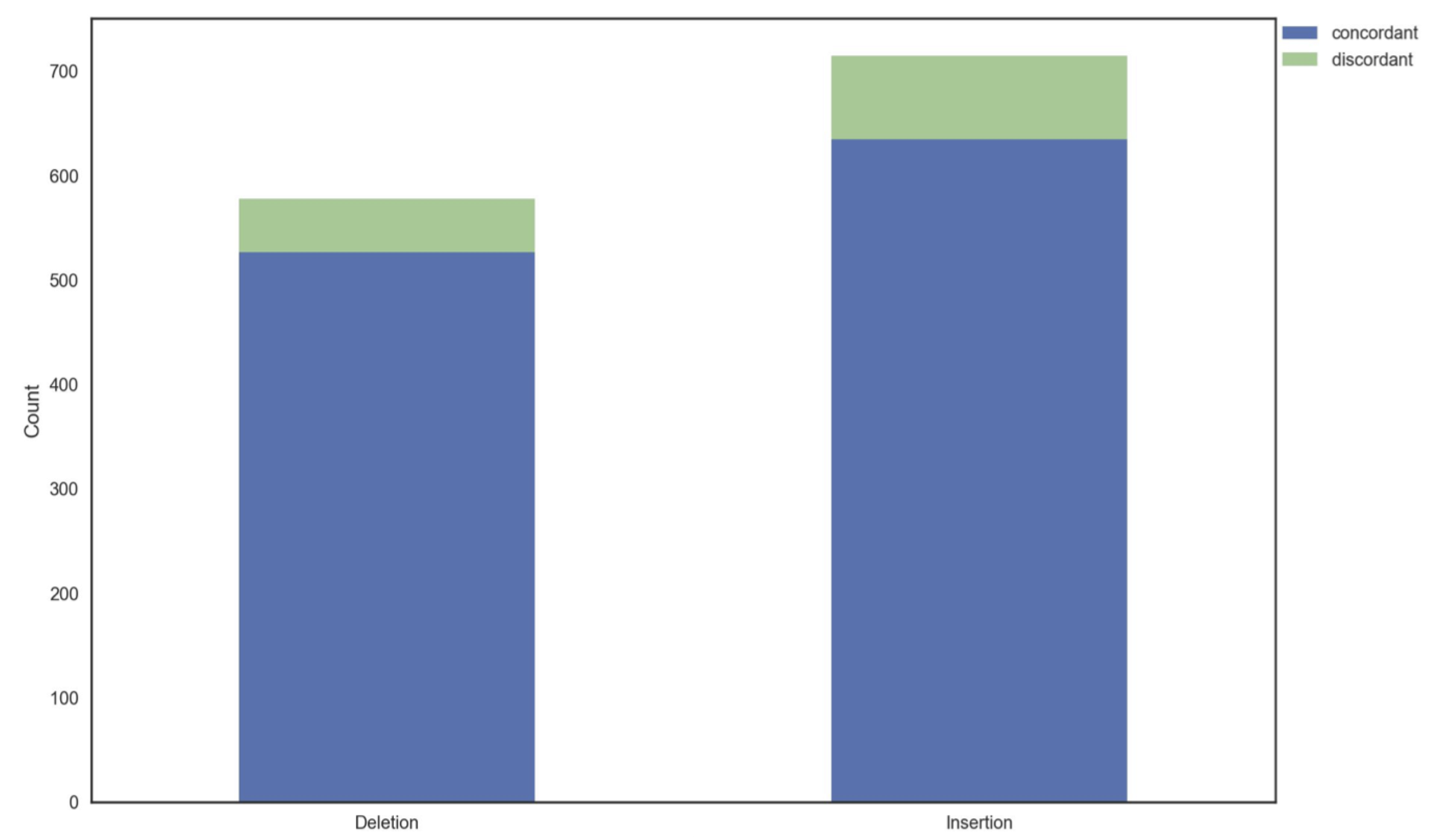

Supplement: S4 Fig — (TIF) [file pcbi.1007933.s004.tif]

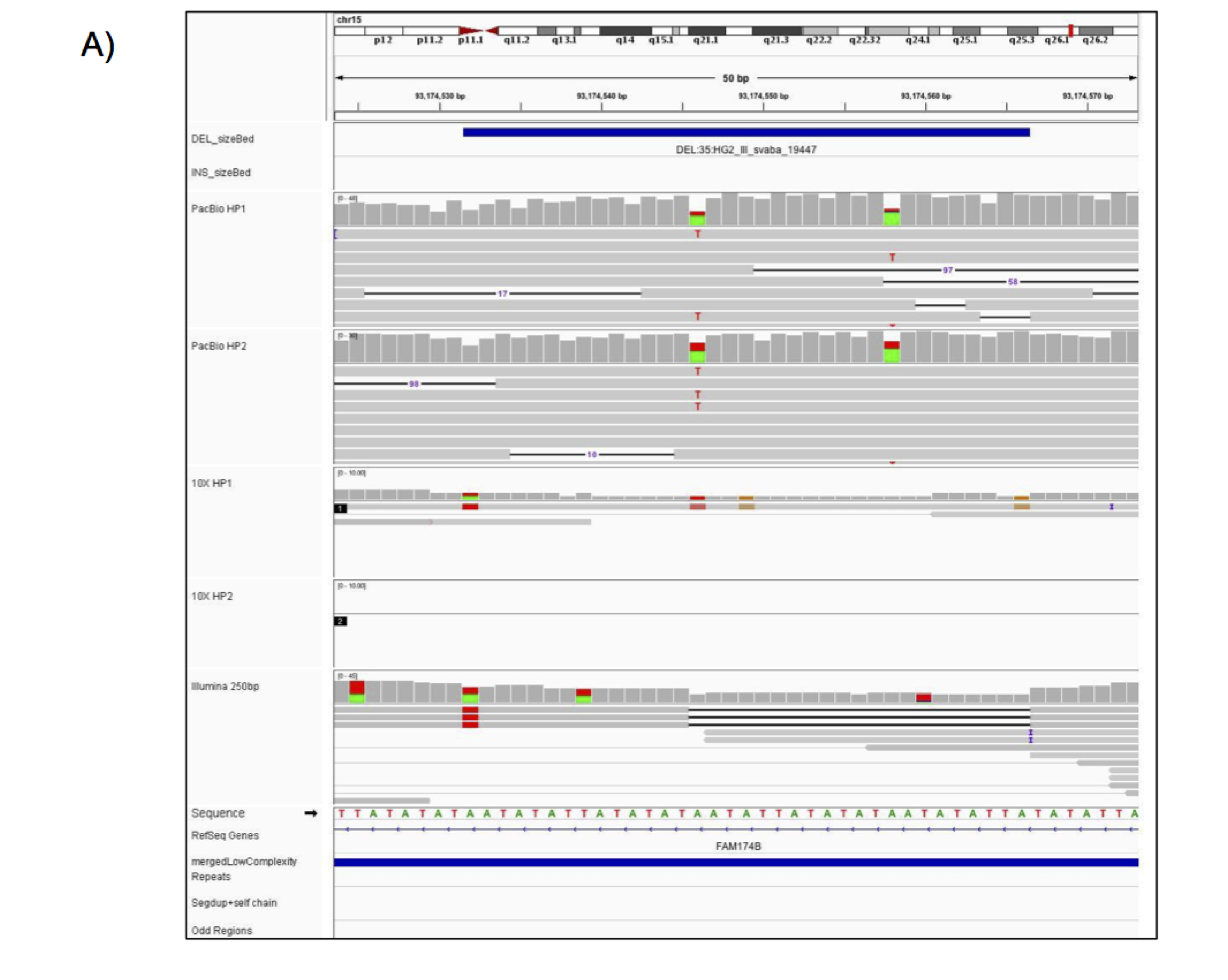

Supplement: S5 Fig — IGV images showing examples of two events that had less than 50% concordance for the label assigned by the curators. (TIF) [file pcbi.1007933.s005.tif]

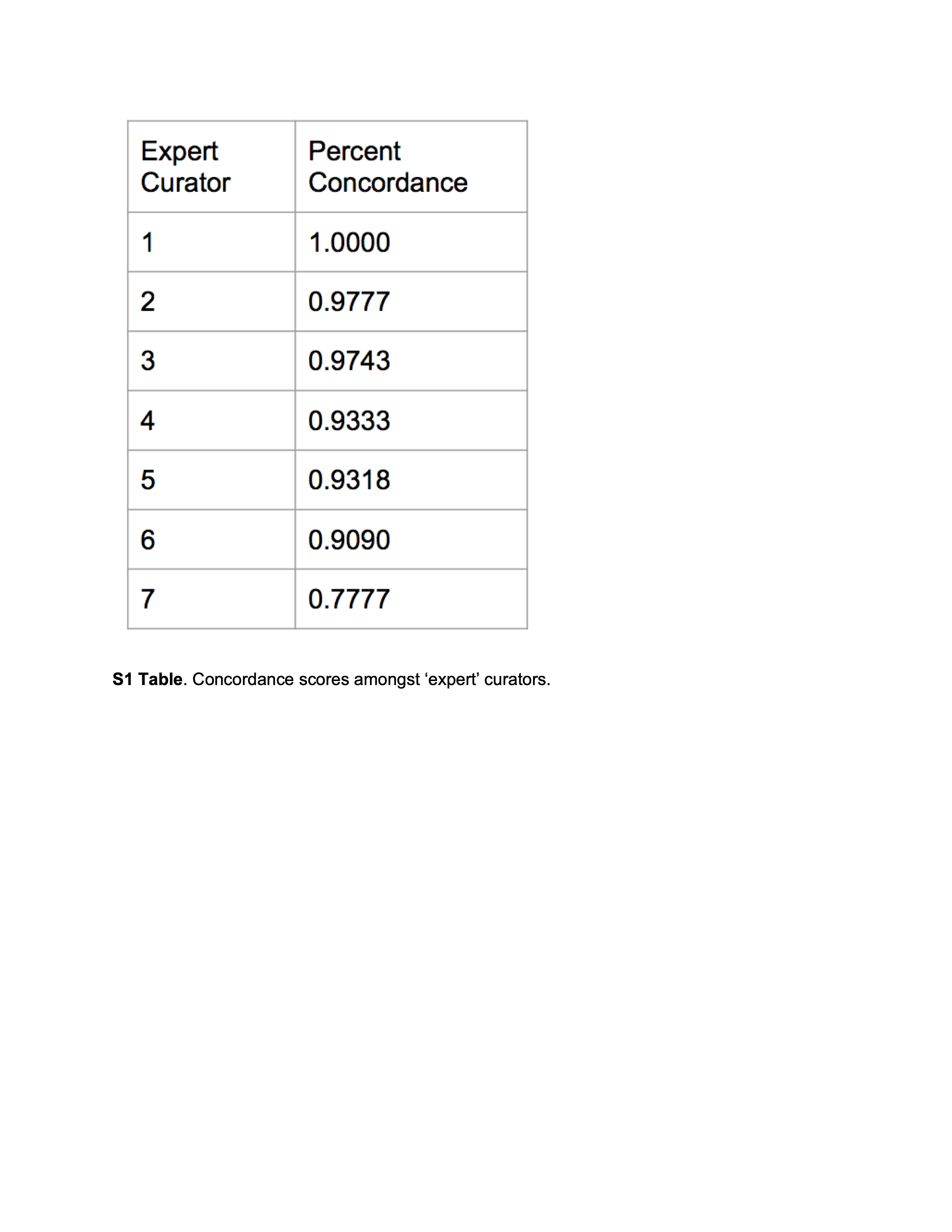

Supplement: S1 Table — (TIF) [file pcbi.1007933.s006.tif]
