# Supplementary material for: A crowdsourced set of curated structural variants for the human genome
Source: PLoS Comput Biol. 2020 Jun 19;16(6):e1007933. doi: 10.1371/journal.pcbi.1007933 (PMC7329145; doi:10.1371/journal.pcbi.1007933)
Supplement: Attachment — Submitted filename: SVCurator Reviewer Response 191126.docx [file pcbi.1007933.s012.docx]

Responses to the Reviewers are in red text.

Reviewer #1: The authors present a tool called SVCurator, which allows users to view images of sequencing reads from multiple different platforms for the same individual and answer questions about the presence of structural variants. The authors used this tool to interrogate putative SVs for HG002 via crowd sourcing and via expert review. The resulting labels were compared across the expert reviewers, the crowd, and compared to the GIAB benchmark calls.

Major Comments:

SVCurator is a valuable tool for visualizing sequence level evidence for structural variants across sequencing platforms. I think the community will benefit from this tool and the results of the study. I have no major suggestions.

Minor Comments:

- I’m curious about the events that the experts commonly agreed upon or disagreed most on. Can you describe these in terms of their qualitative features? Are they similar to what the non-experts agreed or disagreed upon?

-- Thank you for asking about this. We’ve added a paragraph about Fig 5 results: “The responses from Threshold 1 and Threshold 2 top curators were generally concordant for homozygous reference, heterozygous, and homozygous variants, with >80 % concordance among curators for most variants (Fig 5). Threshold 1 top curators were more concordant than Threshold 2 top curators, particularly for insertions, complex variants, and inaccurate variants (Another_Var), but fewer Threshold 1 curators agreed on the assigned labels. Complex events were particularly challenging, having the lowest levels of concordance for top curators within both groups with a mean concordance of 64% and 47% within top curators above Threshold 1 and 2, respectively.” We use this comparison of Threshold 1 and 2 as a more data-driven way to find differences between those with more expertise (Threshold 1) and the broader crowd (Threshold 2).

- In Figure 1, Q1 shows a question about the size range for this variant – how are those ranges generated?

- We have added a sentence to the Fig 1 legend clarifying this “Curators were asked whether a variant exists within ±20% of the predicted size, its genotype, and their confidence in the curated genotype.”

- Did you include any controls (e.g. images of regions where no putative SV call existed)?

- We have added the following text to clarify how control (homozygous reference) regions were included and why we didn’t generate randomly from normal regions of the genome: “Fewer events were homozygous reference than heterozygous or homozygous variant because the events were randomly selected from SV callsets for the son-father-mother trio, and most calls were true SVs in the son. Some events were homozygous reference either because they were FP calls or because they were SVs in the parents that were not inherited by the son, which are more useful controls than randomly generated SVs in the genome. Future evaluations could get a better balance between homozygous reference and true SVs by including more filtered calls from input callsets and by including calls from unrelated individuals.”

- Do you have any information about experts and the crowd make their decisions? Do they weight the data in the same ways or look for particular features?

- We do not have a good way to investigate this given our data, though we agree this could be an interesting aspect to explore in future work.

- Were there any individual SVs (e.g. rather than classes of SVs) that the crowd performed better than the experts? If so, it would be interesting to describe these.

- We were not able to identify any trends in the small number of SVs where the crowd may have performed better than the experts. The biggest advantage noted in the paper is that including the crowd provided more curations of some sites that had few expert curations.

- Figure 3 – These may be easier to read as a violin plot.

- Thank you for the suggestion. We have now changed this to a violin plot.

- Can you explain why the variants in the “another_var” category have such high discordance levels?

- We have added this to the discussion: “In addition, SVs that had inaccurate breakpoints or size estimates were inconsistently classified as Another_Var by curators, particularly curators with less expertise. This inconsistency was likely caused by challenges in interpreting svviz results for inaccurate calls and the lack of evidence within the IGV window, particularly for tandem repeats that extend beyond the window shown. Future efforts may get more accurate curations if they display both a zoomed in image as well as a zoomed out image that fully encompasses any overlapping tandem repeats ”

- When you say that on page 16 that the events were manually inspected – do you mean by the Authors? Or can you explain how this manual inspection is different than what has been described in other places in the manuscript?

- We’ve added a clarifying statement: “In this re-curation by the authors, we used a dynamic IGV session with new data types, including Oxford Nanopore ultralong reads, in addition to the images dispayed in SVCurator.”

- Figure 9 is really helpful. I think it would help the reader understand the methods and definitions of the various categories if you create something like this to include earlier on (e.g. a Figure 1A).

- We’ve now moved this figure up in the manuscript, so that it is now Figure 4.

- Overall I would like to hear more about SVCurator – what parameters or visualizations can be tuned or varied for future studies. I imagine other groups may not have quite so much data on the same sample, but clinical groups, core labs, and SV detection tool developers may be interested in using SVcurator to review data. How flexible will SVCurator be in other studies when not all data types are present? Can you describe the API or general approach for a future user to use this tool?

- We’ve significantly rewritten a paragraph in the discussion to address these important points: “This study suggests that a group of participants can accurately curate SV calls by evaluating a variety of static images from multiple sequencing technologies. In general, isolated heterozygous and homozygous variants and homozygous reference regions were accurately labeled as long as the variant size and type were accurate. A major cause of lower concordance scores were complex events, which were often located in repetitive regions of the genome. One limitation of this experiment was that the IGV images sometimes only displayed part of a large tandem repeat and missed parts of a complex variant, so we recommend that future curation images display a larger region fully encompassing any tandem repeats overlapping the SV. Crowdsourcing studies specifically focused on complex SVs should also likely provide tutorials with several examples of complex events, including where more than one SV is in cis and in trans. In addition, SVs that had inaccurate breakpoints or size estimates were inconsistently classified as Another_Var by curators, particularly confusing curators with less expertise. Ensuring IGV images encompass entire tandem repeats also should help ensure more accurate curations of inaccurately defined SVs. For future studies that may not have as extensive data for curation, we recommend including long reads if curation of SVs in tandem repeats is important and including images from svviz if curation of insertions is important.”

- Are there any limitations of SVCurator? For example, how much depth can it reasonably display? Does it randomly downsample, etc. Are the images static (e.g. screenshots from IGV) or are users able to use IGV features to scroll, group or color reads, etc?

- We’ve added a couple sentences to the discussion about limitations of the current interface and the advantages that a more dynamic interface might provide in future curation efforts: “Enabling curators to dynamically zoom in and out of IGV displays would also make curation of more challenging events like those in tandem repeats or segmental duplications. Dynamic visualizations could also enable users to scroll through higher depth of reads.”

Reviewer #2: The manuscript entitled “SVCurator: A Crowdsourcing app to visualize evidence of structural variants for the human genome” by Dr. Zook and colleagues described an experience of crowdsourcing manual curation of germline structural variations (SV) of a Genome In A Bottole (GIAB) sample by “recruiting” 61 curators to manually review 1235 putative insertions and deletions. The user interface displays all the relevant information generated by diverse technology together on the same page (e.g. PacBio long read, 10X Genomics reads and Illumina reads), which is useful when evaluating as the user can zoom and scroll pretty easily. Overall, having a review strategy of crowed sourced evaluations to generate a reference SV data is an interesting approach and binning the curators into experts (threshold 1 and non-experts (threshold 2) is a good strategy to avoid reducing the quality of the results due to the input from an inexperienced reviewer.

Major issues:

1) The interface appears to be designed for the specific task of reviewing the GIAB samples. There is no way to upload a custom set of SVs. Without an interface for uploading the custom data, it is unclear how the publication of the tool can benefit research community was. The authors also need to provide tutorial on how to upload raw SV data/images, recruit reviewers, and collect and assess results.

- We appreciate the useful feedback that a broadly usable curation platform would be a useful resource for the community. The SVplaudit/plotcritic framework we reference (<https://academic.oup.com/gigascience/article/7/7/giy064/5026174>) is a general-purpose tool like this. Our SVCurator experiment was designed to complement SVplaudit by rendering a variety of types of images as multiple lines of evidence for each putative variant. While SVCurator has a more complex interface and process for generating images, it enabled us to get reliable curations for a broader spectrum of variant types and genome contexts, and to gain an understanding of the challenges of certain types of variants that can guide future development of curation tools (in fact, we’ve been working with the developers of SVplaudit to incorporate some of what we’ve learned into their tool, such as the need to visualize the entire repeat region during curation). We’ve revised the abstract and discussion to better emphasize the most useful aspects of this study, and have changed the title to “A crowdsourced set of curated structural variants for the human genome.”

2) The original SVs were generated from 30 variant callers. Can the author indicate the accuracy and sensitivity of each caller based on the curation consensus?

- Because these variant callers were not optimized for the same tradeoff between precision and recall, and many of the variant callers and technologies have undergone significant improvements since the original SVs were collected, statistics related to accuracy and sensitivity could be misleading. The calls used in curation are also a small subset of all calls and relatively few were from any individual callset. As a more enduring resource, GIAB has worked to develop benchmarks, such as the v0.6 SV benchmark (Zook, J. M. et al. A robust benchmark for germline structural variant detection. bioRxiv 664623 (2019). doi:10.1101/664623), which enable anyone to benchmark performance of their methods of interest as methods evolve.

3) In Discussion, the authors mentioned that the crowdsourcing results could be further validated by PCR. Given the long-read sequencing support, would PCR validation still be required? It would be important to present how many of the crowdsourcing results were corroborated by at least one long-read technology (i.e. PacBio, 10X or long-read library). This information was not presented in Figure 8. It is intriguing to see that a small proportion of the SVs were supported only by <=1 technology (Figure 8A). It will be important to explain why these events are considered real.

- We thank the reviewer for pointing out this result that warranted further investigation. We have remade Figure 8 following the reviewer’s suggestions, and we have also performed in depth manual curation of the 10 sites with no corroboration and 58 events with only 1 technology’s corroboration. We have added the following text:
  “We also used svviz2 to evaluate the curators’ final labeled callset, including variants outside the v0.6 benchmark regions. svviz2 determines whether each read more closely matches the reference allele or the alternate allele or if it is ambiguous. svviz2 generates genotypes [Homozygous Reference, Heterozygous Variant, Homozygous Variant] based on the number of reads that align to the reference and alternate alleles, weighted by their mapping quality scores. We generated svviz2 genotypes from 5 datasets [Illumina 250bp paired end sequencing, Illumina 150bp paired end sequencing, Illumina mate-pair, haplotype-partitioned PacBio and haplotype-partitioned 10x Genomics], and genotypes from each technology were compared to the 879 SVCurator crowdsourced labels that were Homozygous Reference, Heterozygous Variant, or Homozygous Variant. For PacBio and 10x Genomics, calls where either haplotype was heterozygous were conservatively not counted as supporting the label, since almost all the reads on each haplotype should either support the SV or reference allele.
- The crowdsourced label for 91% (804 of the 879 events) were supported by at least 2 technologies ([Fig](https://docs.google.com/presentation/d/1mTvCDkWC40IcPh8NGEKuXSTk8Av5Aw82Lt52DvzYDh0/edit?pli=1#slide=id.g40022f0532_0_111) 8). Upon detailed manual curation with new long read data in a dynamic IGV session, the curators’ genotype was correct for 7 of the 11 events with no svviz genotype support, and the svviz genotypes did not agree mostly due to slight inaccuracy in the SV breakpoints or size. The events incorrectly classified by the curators were mostly complex variants that the curators determined to be simple heterozygous or homozygous variants. There were also 64 events where only 1 technology supported the crowdsourced label, 40 only by PacBio and 16 only by mate-pair (Supplementary Table 2). We performed detailed manual curation of 10 of the sites supported only by PacBio, and the curators were correct in 8 of them, called one event a simple deletion when in fact it was complex due to a 500bp insertion on the other haplotype, and called one event homozygous variant even though it was slightly larger than the predicted size range. We performed detailed manual curation of 10 of the sites supported only by Illumina mate-pair, and the curators were correct for 4 of them, 3 should have been called complex due to different variants on opposite haplotypes, and 2 were unclear due to alpha satellites near the centromere, and 1 was likely a deletion in a larger duplicated region. Upon curation of all 6 sites only supported by 250bp illumina reads, 5 were correctly called 20-100bp indels in TRs between the 150bp and 250bp illumina read lengths, and one should have been called Another_Var since it was larger, though this was not obvious in the static images displayed in SVCurator. These results further support the accuracy of the crowdsourced labeled events, including those outside the v0.6 benchmark regions, and also points to causes of the small number of curators’ errors due to complex variants or imprecisely called variants in repetitive regions of the genome.”
- We also agree that PCR validation is generally unlikely to provide much additional confidence beyond manual curation of the extensive short, linked, and long read data available for the GIAB samples, and we have removed this statement.

4a) The quality of the manuscript needs to be improved. The text label of Figure 1 is not readable, Figure 7 was of poor quality; Figure 8 used different width to show the bar plot and Figure 9 was first referred on page 15, ahead of Figure 6.

- We have revised the figures to make them higher quality and fixed the order

4b) A number of paragraphs describe the performance of a specific group of curators working with this specific dataset. This content may need to be removed as it is of little scientific interest and yields little insight.

- We now clarify that the purpose of these results are to select “top curators” to use in forming the final curated set. We’ve also added a statement to the discussion “While all of the metrics are specific to this group of curators, we learned several things that could improve future curation studies.”

4c)The phrase "python flask web based app" appears throughout the manuscript which does not make sense for people who are not in the field of software development.

- We have modified this to use less jargon

Minor issues

1a) The “help” tab on the web page is broken, and there is another dead link on the page which raises the concern that an additional image isn’t being rendered.

- Thank you for noting this, the help tab now points to the tutorial page

1b) Also, I’m not seeing any that shows where the soft clipped bases are located, which can be important for SV review. They do show realignment, but without checking the softclip orientations and locations, this can be misleading.

- We have added a clarification to the Methods “Soft-clipping was shown by coloring ends of clipped reads but clipped bases were not shown in order to make the view cleaner.”

**Have all data underlying the figures and results presented in the manuscript been provided?**

Large-scale datasets should be made available via a public repository as described in the *PLOS Computational Biology* [data availability policy](https://gcc01.safelinks.protection.outlook.com/?url=http%3A%2F%2Fjournals.plos.org%2Fploscompbiol%2Fs%2Fdata-availability&data=02%7C01%7Cjustin.zook%40nist.gov%7C73cc45bda1e74af467cf08d772946be1%7C2ab5d82fd8fa4797a93e054655c61dec%7C1%7C1%7C637103854098133896&sdata=HcYgKfz4kxGsPzYKgjuM6eEr8fYX83YALhjMJD6GkNE%3D&reserved=0), and numerical data that underlies graphs or summary statistics should be provided in spreadsheet form as supporting information.

Reviewer #1: Yes

Reviewer #2: Yes

PLOS authors have the option to publish the peer review history of their article ([what does this mean?](https://gcc01.safelinks.protection.outlook.com/?url=https%3A%2F%2Fjournals.plos.org%2Fploscompbiol%2Fs%2Feditorial-and-peer-review-process%23loc-peer-review-history&data=02%7C01%7Cjustin.zook%40nist.gov%7C73cc45bda1e74af467cf08d772946be1%7C2ab5d82fd8fa4797a93e054655c61dec%7C1%7C1%7C637103854098143893&sdata=rF9QwODNmrQgKQ4thRsza%2BrLglB5FNLFlsGtch6gtDg%3D&reserved=0)). If published, this will include your full peer review and any attached files.

If you choose “no”, your identity will remain anonymous but your review may still be made public.

**Do you want your identity to be public for this peer review?** For information about this choice, including consent withdrawal, please see our [Privacy Policy](https://gcc01.safelinks.protection.outlook.com/?url=https%3A%2F%2Fwww.plos.org%2Fprivacy-policy&data=02%7C01%7Cjustin.zook%40nist.gov%7C73cc45bda1e74af467cf08d772946be1%7C2ab5d82fd8fa4797a93e054655c61dec%7C1%7C1%7C637103854098143893&sdata=UGVElWA7M7gmvc14Strmr4TwcenKnrak2ZUHSlIOu%2BM%3D&reserved=0).

Reviewer #1: No

Reviewer #2: No
